# Running in the FAMILY: understanding and predicting the intergenerational transmission of mental illness

**DOI:** 10.1007/s00787-024-02423-9

**Published:** 2024-04-13

**Authors:** Lisanne A. E. M. van Houtum, William F. C. Baaré, Christian F. Beckmann, Josefina Castro-Fornieles, Charlotte A. M. Cecil, Juliane Dittrich, Bjørn H. Ebdrup, Jörg M. Fegert, Alexandra Havdahl, Manon H. J. Hillegers, Raffael Kalisch, Steven A. Kushner, Isabelle M. Mansuy, Signe Mežinska, Carmen Moreno, Ryan L. Muetzel, Alexander Neumann, Merete Nordentoft, Jean-Baptiste Pingault, Martin Preisig, Andrea Raballo, John Saunders, Emma Sprooten, Gisela Sugranyes, Henning Tiemeier, Geeske M. van Woerden, Caroline L. Vandeleur, Neeltje E. M. van Haren

**Affiliations:** 1 Department of Child and Adolescent Psychiatry/Psychology, Erasmus MC, University Medical Centre–Sophia, Rotterdam, The Netherlands; 2 Danish Research Centre for Magnetic Resonance, Centre for Functional and Diagnostic Imaging and Research, Copenhagen University Hospital-Amager and Hvidovre, Copenhagen, Denmark; 3 Centre for Functional MRI of the Brain, Wellcome Centre for Integrative Neuroimaging, Nuffield Department of Clinical Neurosciences, University of Oxford, Oxford, UK; 4 Department of Cognitive Neuroscience, Radboud University Medical Centre, Nijmegen, the Netherlands; 5 Donders Institute for Brain, Cognition and Behaviour, Radboud University Nijmegen, Nijmegen, the Netherlands; 6 Department of Child and Adolescent Psychiatry and Psychology, 2021SGR01319, Institut Clinic de Neurociències, Hospital Clínic de Barcelona, FCRB-IDIBAPS, Centro de Investigación Biomédica en Red de Salud Mental (CIBERSAM), Department of Medicine, Institute of Neuroscience, University of Barcelona, Barcelona, Spain; 7 Department of Epidemiology, Erasmus MC, University Medical Centre Rotterdam, Rotterdam, the Netherlands; 8 Concentris Research Management Gmbh, Fürstenfeldbruck, Germany; 9 Center for Neuropsychiatric Schizophrenia Research and Centre for Clinical Intervention and Neuropsychiatric Schizophrenia Research, Mental Health Centre Glostrup, University of Copenhagen, Glostrup, Denmark; 10 Department of Clinical Medicine, Faculty of Health and Medical Sciences, University of Copenhagen, Copenhagen, Denmark; 11 President European Society for Child and Adolescent Psychiatry (ESCAP), Brussels, Belgium; 12 Department of Child and Adolescent Psychiatry/Psychotherapy, University Hospital Ulm, Ulm, Germany; 13 PsychGen Centre for Genetic Epidemiology and Mental Health, Norwegian Institute of Public Health, Oslo, Norway; 14 PROMENTA Research Centre, Department of Psychology, University of Oslo, Oslo, Norway; 15 Nic Waals Institute, Lovisenberg Diaconal Hospital, Oslo, Norway; 16 Leibniz Institute for Resilience Research, Mainz, Germany; 17 Neuroimaging Center (NIC), Focus Program Translational Neuroscience (FTN), Johannes Gutenberg University Medical Center, Mainz, Germany; 18 Department of Psychiatry, Erasmus MC, University Medical Centre Rotterdam, Rotterdam, The Netherlands; 19 Laboratory of Neuroepigenetics, Medical Faculty, Brain Research Institute, Department of Health Science and Technology of ETH, University of Zurich and Institute for Neuroscience, Zurich, Switzerland; 20 Zurich Neuroscience Centre, ETH and University of Zurich, Zurich, Switzerland; 21 Institute of Clinical and Preventive Medicine, University of Latvia, Riga, Latvia; 22 Department of Child and Adolescent Psychiatry, Institute of Psychiatry and Mental Health, Hospital General Universitario Gregorio Marañón, IiSGM, CIBERSAM, ISCIII, School of Medicine, Universidad Complutense, Madrid, Spain; 23 Department of Radiology and Nuclear Medicine, Erasmus University Medical Centre, Rotterdam, The Netherlands; 24 The Lundbeck Foundation Initiative for Integrative Psychiatric Research, Aarhus, Denmark; 25 Copenhagen Research Centre for Mental Health, Mental Health Centre Copenhagen, Copenhagen University Hospital, Copenhagen, Denmark; 26 Social, Genetic and Developmental Psychiatry Centre, Institute of Psychiatry, Psychology and Neuroscience, King’s College London, London, UK; 27 Department of Clinical, Educational and Health Psychology, University College London, London, UK; 28 Psychiatric Epidemiology and Psychopathology Research Centre, Department of Psychiatry, Lausanne University Hospital and University of Lausanne, Lausanne, Switzerland; 29 Public Health Division, Department of Health and Social Care, Cantonal Socio-Psychiatric Organization, Repubblica e Cantone Ticino, Mendrisio, Switzerland; 30 Chair of Psychiatry, Faculty of Biomedical Sciences, Università Della Svizzera Italiana, Lugano, Switzerland; 31 Executive Director European Federation of Associations of Families of People with Mental Illness (EUFAMI), Louvain, Belgium; 32 Department of Human Genetics, Radboud University Medical Centre, Nijmegen, The Netherlands; 33 Department of Social and Behavioural Sciences, Harvard T.H. Chan School of Public Health, Boston, MA USA; 34 Department of Neuroscience, Erasmus University Medical Centre, Rotterdam, The Netherlands; 35 ENCORE Expertise Center for Neurodevelopmental Disorders, Erasmus University Medical Centre, Rotterdam, The Netherlands; 36 Department of Clinical Genetics, Erasmus University Medical Centre, Rotterdam, The Netherlands

**Keywords:** Intergenerational transmission, Mental illness, Family, Offspring, Risk prediction, Resilience

## Abstract

Over 50% of children with a parent with severe mental illness will develop mental illness by early adulthood. However, intergenerational transmission of risk for mental illness in one’s children is insufficiently considered in clinical practice, nor is it sufficiently utilised into diagnostics and care for children of ill parents. This leads to delays in diagnosing young offspring and missed opportunities for protective actions and resilience strengthening. Prior twin, family, and adoption studies suggest that the aetiology of mental illness is governed by a complex interplay of genetic and environmental factors, potentially mediated by changes in epigenetic programming and brain development. However, how these factors ultimately materialise into mental disorders remains unclear. Here, we present the FAMILY consortium, an interdisciplinary, multimodal (e.g., (epi)genetics, neuroimaging, environment, behaviour), multilevel (e.g., individual-level, family-level), and multisite study funded by a European Union Horizon-Staying-Healthy-2021 grant. FAMILY focuses on understanding and prediction of intergenerational transmission of mental illness, using genetically informed causal inference, multimodal normative prediction, and animal modelling. Moreover, FAMILY applies methods from social sciences to map social and ethical consequences of risk prediction to prepare clinical practice for future implementation. FAMILY aims to deliver: (i) new discoveries clarifying the aetiology of mental illness and the process of resilience, thereby providing new targets for prevention and intervention studies; (ii) a risk prediction model within a normative modelling framework to predict who is at risk for developing mental illness; and (iii) insight into social and ethical issues related to risk prediction to inform clinical guidelines.

## Introduction

A family history of severe mental illness is a well-known, important risk factor for developing mental health problems. Over 50% of children with a parent with severe mental illness will develop a mental disorder by early adulthood [1, 2], demonstrating a tangible transfer of risk from affected parents to offspring. However, intergenerational transmission of risk for mental illness in offspring of patients is insufficiently considered in clinical practice [3]. Healthcare systems do not sufficiently utilise (and in most cases substantially neglect) family history of mental illness into diagnostics and care of offspring of parents with a mental health disorder, leading to delays in diagnosing young offspring and missing opportunities for protective actions and resilience strengthening [4, 5]. Although parents with mental illness are often concerned that their disorder may impact the wellbeing of their children, due to genetic risk or possible parenting difficulties [6], healthcare professionals seldomly discuss their patient’s worries, parenting role and style [4, 7]. Critically, a family-based approach to manage mental disorders is hampered by the generally strong focus on individual recovery and clinical management and the gap between child and adolescent and adult mental health services [8]. Currently, healthcare professionals are in need for tools or guidelines, or even a change in the system, to pay adequate consideration to these factors. Together with fear of stigma, these issues form obstacles for both parents and offspring to seek professional help [9].

Twin, family, and adoption studies suggest that the aetiology of mental illness is governed by a complex interplay between substantial genetic factors (heritability estimates between 0.4 and 0.8 [10]) and environmental factors. Such gene-environment interplay involves many common susceptibility genes with small effects, few rare genetic variants with larger effects, and a variety of environmental risk factors [11]. Biological mechanisms, like epigenetic processes (DNA methylation (DNAm) and non-coding RNAs [12]) and brain development, may mediate how genetic and environmental factors ultimately materialise into mental disorders along the lifespan. Importantly, the increased familial risk of mental illness can be dampened by resilience factors that themselves can be of genetic or environmental origin, including supportive parenting style or social support [13].

Despite ample evidence that mental illness runs in families [2], how and when risk for mental illness is passed from parents to offspring is still poorly understood, which hinders the identification of new targets for prevention and treatment strategies. The understanding of intergenerational transmission of risk mechanisms could be advanced by identifying the underlying environmental and genetic risk factors and mediating epigenetic and neural mechanisms, and when these factors operate, e.g. during foetal development, early childhood, adolescence, and/or into adulthood. Concurrently, resilience factors counteracting an existing risk and their mechanisms of action need to be identified, taking advantage of the fact that a substantial proportion of individuals having a parent with mental illness do not develop disorders themselves [14]. Therefore, FAMILY’s first aim is to advance our understanding of the aetiology of familial risk for and resilience to mental illness, thereby providing new targets for prevention and intervention studies, to break the intergenerational cycle of mental illness.

Risk prediction models for mental disorders critically depend on the suitable identification and conceptualization of relevant variables, and poor, sub-optimal risk prediction obviously limits the early identification of who could benefit most from preventive actions that aim to reduce risk or promote resilience. Therefore, by studying the intergenerational transmission of risk of mental illness, reliable quantitative in addition to qualitative metrics in parents and their offspring may be obtained, informative of the likelihood of offspring to develop mental illness. Such metrics should include clinical, behavioural, environmental, as well as biological factors. Thus, characteristics of parental illness, parental and offspring genetic, epigenetic, and neuroanatomical markers, but also parent- and/or child-specific information, such as behaviour, personality, life experiences, and social factors (e.g., social class or social networks), can contribute to the prediction of risk for mental illness in offspring. Crucially, such risk and resilience markers that play a role in risk prediction within families may be equally informative in youth without a known family history of mental illness.

Risk prediction models for mental disorders have been developed, but currently have severe limitations: (i) they are restricted to individual-level information and do not take parental and family information into account; (ii) they predict outcomes primarily based on information from emerging or subclinical psychiatric symptoms and level of functioning [15] and thus at a relatively late stage before disorder manifestation, leaving little to no time to intervene; (iii) they have mainly been developed for psychotic disorders [16]; (iv) they focus largely on a single biomarker modality (e.g. genetics, epigenetics, or neuroimaging) without biomarker integration; (v) they include only risk factors and do not consider resilience factors; and (vi) they mostly have not been replicated or validated in independent samples and are thus not yet translatable to clinical practice [17]. Our second aim is to construct and test a prediction model that overcomes these shortcomings and is maximised for accuracy, such that the [joint] contributions of relevant factors to increased risk are known. We aim to predict who will develop symptoms or meet the diagnostic criteria for a mental disorder later in life, and apply the prediction models to the general population and to those at high familial risk because of having a parent with a severe mental disorder.

Predicting the risk for mental disorders in children of affected parents would radically change the clinical approach to mental illness. However, implementation of (family-based) risk prediction models in health services, once fully validated, requires fundamental changes in clinical practice and thorough preparation of all relevant stakeholders, including policymakers. Critically, ethical and social consequences need careful attention and appraisal, such as the risk of self-fulfilling prophecies [18], the concept of neurodiversity [19], the right not to know [20, 21], the risk of stigma [22, 23], and the use of artificial intelligence in risk prediction and data sharing [24]. Therefore, our third aim is to provide insights into social and ethical issues related to risk prediction, to inform ethical and clinical guidelines.

**Fig. 1:**
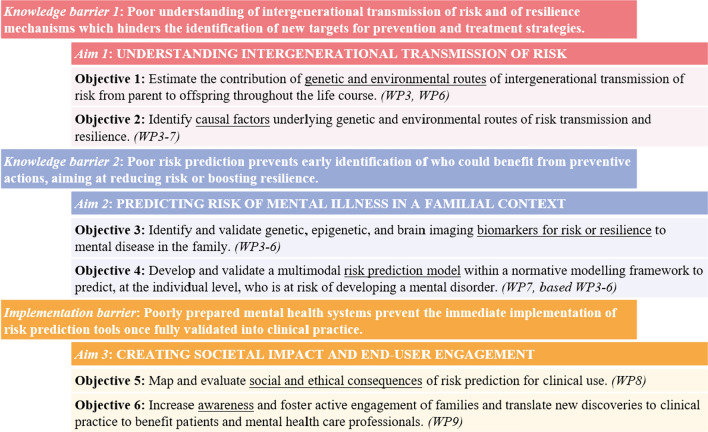
Knowledge and implementation barriers are translated into FAMILY's three aims with six key objectives

Here, we present FAMILY, a large-scale multidisciplinary initiative to predict the risk for mental disorders in children of affected parents and in the general population and to better understand the mechanisms of intergenerational transmission of mental illness, using genetically informed causal inference and novel multimodal normative prediction models. FAMILY’s three aims are translated into six key objectives (Fig. 1). FAMILY brings together a consortium of experts in psychiatry, developmental psychology, social sciences, (epi)genetics, neuroscience, data science, and bioethics. In total, 15 institutions across Europe and one institution in the United States collaborate (Table 1). ESCAP and EUFAMI are our societal partners, crucial for knowledge dissemination and active participation of families, healthcare professionals, and policymakers.

**Table 1:** List of beneficiary and affiliated partners who collaborate in the FAMILY consortium

Participant number	Participant organization name (acronym)	Participant country
Beneficiary partners		
1 (coordinator)	ERASMUS UNIVERSITAIR MEDISCH CENTRUM ROTTERDAM (EMC)	Netherlands
2	STICHTING RADBOUD UNIVERSITAIR MEDISCH CENTRUM (RUMC)	Netherlands
3	LEIBNIZ-INSTITUT FUR RESILIENZFORSCHUNG (LIR)	Germany
4	LATVIJAS UNIVERSITATE (LU)	Latvia
5	FOLKEHELSEINSTITUTTET—NORWEGIAN INSTITUTE OF PUBLIC HEALTH (NIPH)	Norway
6	FUNDACIO CLINIC PER A LA RECERCA BIOMEDICA (FCRB)	Spain
7	HARVARD GLOBAL RESEARCH AND SUPPORT SERVICES INC. (HARVARD GLOBAL)	United States
8	CONCENTRIS RESEARCH MANAGEMENT GMBH (concentris)	Germany
9	FUNDACION PARA LA INVESTIGACION BIOMEDICA DEL HOSPITAL GREGORIO MARANON (FIBHGM)	Spain
10	REGION HOVEDSTADEN (RegionH)	Denmark
11	EUROPEAN SOCIETY FOR CHILD AND ADOLESCENT PSYCHIATRY (ESCAP)	Belgium
12	EUROPESE FEDERATIE VAN FAMILIEVERENIGINGEN VAN PSYCHIATRISCH ZIEKE PERSONEN IVZW (EUFAMI)	Belgium
Affiliated partners		
13	UNIVERSITY COLLEGE LONDON (UCL)	United Kingdom
14	UNIVERSITAT ZURICH (UZH)	Switzerland
15	CENTRE HOSPITALIER UNIVERSITAIRE VAUDOIS (CHUV)	Switzerland
16	UNIVERSITA DELLA SVIZZERA ITALIANA (USI)	Switzerland

FAMILY will specifically focus on the risk for psychiatric symptoms and diagnoses in children of parents with a diagnosis in the mood-psychosis spectrum (i.e., schizophrenia, bipolar disorder or depression)*.* Although current diagnoses of mood and psychosis spectrum disorders are categorical, as exemplified by the DSM-5 [25], many difficulties come with such categorical approaches. For example, there is a high percentage of comorbidities, patients with different diagnoses may use the same drug, there is a lack of validated animal models for diagnoses, and a lack of diagnostic specificity in genetic [26] and neuroimaging markers [27, 28]. In FAMILY, we will take a cross-disorder and dimensional approach [29], by aiming to predict the risk of homotypic and heterotypic transmission of (i) clinically relevant symptoms and (ii) diagnoses for an individual. As there is no clinical alternative (yet) to the current classification systems and categorical diagnoses remain relevant for communication and treatment decision making, prediction models are required to also estimate risk of diagnoses. Despite its focus on the mood-psychosis spectrum, FAMILY will deliver a generic approach that can be implemented to build prediction models for other types of psychiatric symptoms and diagnoses as well.

## Methods

### Work package framework

**Fig. 2:**
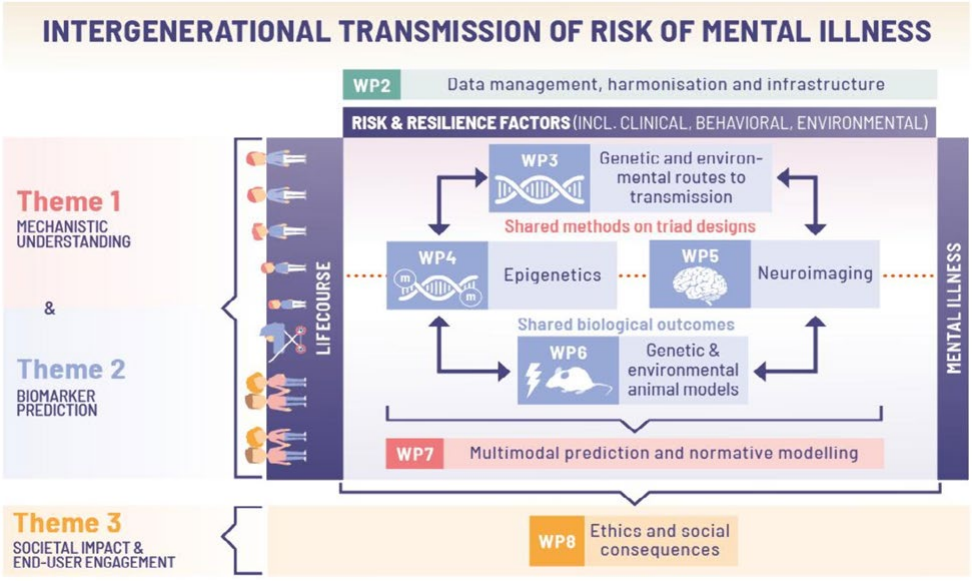
FAMILY’s work package framework

FAMILY puts in place six empirical work packages (WPs3-8) to reach its objectives (Fig. 2). This WP framework is characterised by strong interconnections and synergistic methodological approaches between the proposed research in objectives 1–4 (WPs3-7). WPs3-5 work with human data and each focus on their own biological level, i.e., the genome (e.g., polygenic risk; WP3), epigenome (e.g., DNAm, microRNA; WP4), and the brain (e.g., magnetic resonance imaging (MRI); WP5). Information from these data domains may act as predictors, mediators, or moderators of intergenerational transmission of risk. Furthermore, to increase mechanistic understanding of the role of intergenerational transmission, WP6 will leverage two unique animal models: one where maternal disorder-like behaviour originates from postnatal severe stress, and one where parental disorder-like behaviour results from purely genetic risk. As in the human studies (i.e., WPs3-5), effects on behaviour and biology in the offspring will be investigated. WPs4-5 will share biological readouts with WP6, i.e., epigenetic and neuroimaging markers to allow translation between animal and human findings. Overall, results from WPs3-5 and mechanistic information from WP6 will be integrated and validated in humans in WP7. WP7’s multilevel and multimodal integration is not limited to the biological domains but also includes environmental and behavioural factors. Ultimately, WP7 will develop an individualised risk or resilience prediction model of mental health problems using multimodal normative prediction approaches informed by causal pathways, allowing for prediction systems, which also provide etiological insights. Lastly, to address objectives 5 and 6, WP8 will utilise methods from the social sciences to map social and ethical consequences of risk prediction models as a first step to prepare clinical practice for its future implementation.

### Population and familial high-risk cohorts

FAMILY leverages existing longitudinal general population cohorts and familial high-risk offspring cohorts (with at least one parent with a confirmed diagnosis in the mood-psychosis spectrum) spanning childhood to adulthood with available data from children as well as their mother and/or father (i.e., triads and dyads). Both these general population and familial high-risk offspring cohorts include longitudinal assessments of clinical, behavioural, environmental, genetic, and neuroimaging information (Table 2). These already rich data resources will be expanded by (i) generating circulating microRNA (miRNA) profiles at birth from cord blood plasma in 1600 children from Generation R as an additional type of epigenetic process on top of more commonly investigated DNAm; and (ii) collecting parental brain imaging and blood or saliva samples (for those where samples are missing or of poor quality) in familial high-risk cohorts to obtain triad or dyad MRI and genetic datasets of child, mother and/or father (Table 2).

**Table 2:** Overview of cohorts available to FAMILY

Name	Partner	*n*	*n* Genetics	*n* DNAm	*n* Neuroimaging	WP
Population studies						
GenR/ORACLE	EMC	9700	5900 families	2500 children^a^	2200 families 5500 children	3, 4, 5, 7
COPSYCH	RegionH	700	700 families	0	480 children	5
ALSPAC	On request	15,500	8300 families	1000 families	960 children	3, 4
MoBa	NIPH	114,500	80,000 families (trios/duos)^b^	> 3000 families + 1,800 children	0	3, 4
MCS	UCL	18,800	8800 families	0	0	3
UK Biobank	On request	500,000 adults	40,000 families (6000 parent/offspring)	0	100,000 adults (currently 46,900)	3, 7
ABCD	On request	11,800	9600 children (incl. 860 twin pairs)	0	11,700 children (incl. 860 twin pairs)	5, 7
HCP	On request	1200	1100 adults (incl. 450 siblings)	0	1100 children (incl. 450 siblings)	7
PNC	On request	9500	9500 children	0	1400 children	7
Familial high-risk offspring cohorts						
DBSOS	EMC	208	150 families^c^	0	150 families^c^	3, 5, 7
DBOS	EMC	140	69 families	0	0	3, 7
MARIO	EMC	500^d^	250 families^d^	0	0	3, 7
BASYS	FCRB	277	185 families^e^	0	185 families^e^	3, 5, 7
BASYS	FIBHGM	106	55 families^f^	0	55 families^f^	3, 5, 7
LG	CHUV	159	100 families^g^	0	60 families^g^	3, 5, 7
VIA	RegionH	522	522 families	300 offspring	371 offspring	3, 4, 5, 7

^a^We expect to additionally profile circulating miRNA in *n* = 1600 children

^b^The sample includes siblings

^c^Estimates based on collecting additional blood and MRI in *n* =  ~ 450 parents and offspring

^d^Estimates, data collection is ongoing

^e^Estimates based on collecting blood and MRI in *n* =  ~ 370 parents

^f^Estimates based on collecting blood and MRI in *n* =  ~ 60 parents

^g^Estimates based on collecting blood and MRI in *n* =  ~ 150 parents and offspring

DNAm, DNA methylation; WP, work package

### Work packages

#### WP3: Genetically informed designs to disentangle routes of transmission

**Fig. 3:**
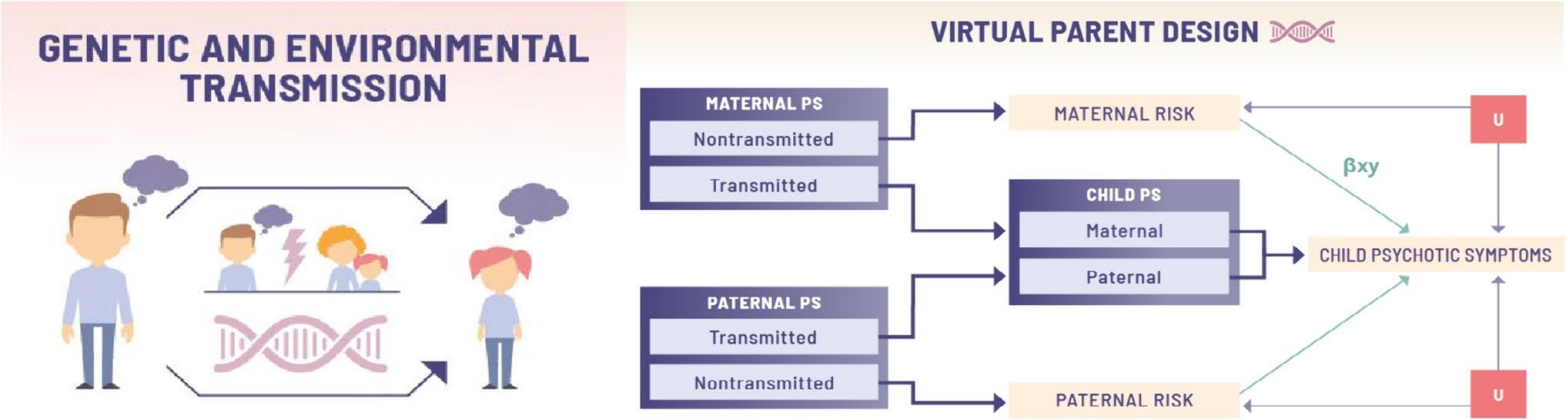
The virtual parent design: genetic and environmental transmission in an intergenerational context. PS, psychotic symptoms; U, unobserved confounders

In WP3, using DNA-variants from triads (father, mother, child) we aim to provide insight into genetic and environmental routes of risk transmission from parents to offspring. In an intergenerational context (Fig. 3), a risk factor, e.g., parental psychotic symptoms, can be associated with offspring psychotic symptoms (i) via the transmission of parental genes (entirely genetic transmission; bottom arrow); and (ii) via an environmentally transmitted effect (top arrow), where parental psychotic symptoms play a role in the emergence of offspring's symptoms. This relates to the concept of ‘genetic nurture’ [30], i.e., the environmentally mediated effect of parental genetics, reflecting the fact that the offspring's nurturing environment is partially shaped by their parents' genetic risk. For example, a risk variant increasing symptoms of psychosis in the parent can impact offspring symptoms via disrupting parenting abilities even when the child does not inherit this variant.

Genetic variants measured throughout the genome can be summarised in a polygenic score, i.e., an individual-level score that captures genetic contribution for a given trait. The *virtual parent design* [30–34] splits polygenic scores corresponding to a particular disorder into transmitted and non-transmitted polygenic scores (Fig. 3). The transmitted scores from both parents form the child’s polygenic score. The path from the parental polygenic score to offspring psychotic symptoms via the offspring polygenic scores corresponds to genetic transmission. Conversely, the path from the non-transmitted polygenic score to the offspring outcome via parental behaviour corresponds to genetic nurture. This genetic nurture path must be environmentally mediated, as genetic transmission is already accounted for.

In WP3, we will develop and apply cutting-edge approaches to estimate genetic nurture effects and identify risk and resilience factors underlying intergenerational transmission of risk of mental illness. That is, genetic nurture and genetic transmission effects will be estimated from polygenic scores from theoretically-based candidate parental risk and resilience factors for offspring psychotic and mood symptoms, including polygenic scores for depression, bipolar disorder and schizophrenia, other psychiatric disorders, and neurodevelopmental conditions, as well as parental education, lifestyle, and physical health. Polygenic scores will also be constructed based on the main independent components from thousands of genome-wide association studies (GWAS) of brain-related traits from WP7. Methodological extensions, such as Mendelian Randomisation (MR), can test whether such factors have a causal effect on child outcomes. MR exploits non-transmitted parental variants related to a risk factor as instruments to assess the causal effect of this risk factor on the outcome. In intergenerational MR, the non-transmitted variants associated to parental risk can be used to estimate the effect of parental risk on offspring outcome (path *βxy* in Fig. 3) independently of unobserved confounders of this association (*U* in Fig. 3) [35]. Risk or resilience factors for which a genetic nurture effect is detected will be followed up by MR analyses to test causal relationships directly. Findings in large population cohorts will be tested in familial high-risk offspring cohorts.

### WP4: Epigenetic routes of transmission

In WP4, we aim to characterise the relevance of epigenetic processes for the transmission of psychiatric risk. Epigenetic processes have been hypothesised to underlie transmission of risk of mental illness based on observations that (i) epigenetic patterns are influenced by both genetic and environmental factors, starting in utero [36]; (ii) they play an essential role in normative development, including brain maturation and function [37]; and (iii) they are associated with numerous psychiatric disorders, including psychosis and mood disorders [38, 39]. DNAm has by far been the most widely examined epigenetic process, showing huge promise as a biological marker for disorder prediction, early detection, and risk stratification [40–43]. However, evidence for a role of DNAm in intergenerational transmission of mental illness remains scarce. In contrast to DNAm, circulating miRNAs have received less attention in humans but are increasingly implicated in intergenerational transmission based on animal studies. For example, rodent and *C. elegans* models have identified miRNAs mediating the effect of parental preconceptional environmental exposures (e.g., starvation, chemical, and stress-related exposures) on offspring health outcomes [44, 45]. Currently, no large-scale data of circulating miRNAs exists in early life, which can be linked to parental exposures and offspring outcomes [46]. Importantly, we are unaware of any largescale study featuring paired samples of *both* DNAm and miRNA profiles, limiting insights into which processes contribute most to intergenerational transmission.

In WP4, we will apply innovative approaches, including the genetic triad design (from WP3) and machine learning methods. Analyses will be based on data from Generation R [47], ALSPAC [48], and MoBa [49] (Table 2) and will primarily focus on epigenetic patterns at birth, linking parental mental illness to offspring mental health outcomes, pre-symptom manifestation. Relevant markers will then be tested for stability vs change across development in studies with long follow-up using the available repeated epigenetic assessments. The generated results will be replicated using data from the Pregnancy and Childhood Epigenetics (PACE) consortium [50] and tested in the RegionH-VIA familial high-risk offspring cohort (Table 2).

### WP5: Neuroimaging routes of transmission

Within WP5, we aim to investigate the role of brain structure and function in the transmission of risk of mental illness from parents to offspring. Brain structural and functional metrics and their developmental trajectories have been hypothesised to underlie the development of mental illness, based on observations that (i) brain metrics are influenced by genetic and familial environmental factors [51]; (ii) they play an essential role in typical and atypical development during adolescence [52]; (iii) brain alterations are associated with numerous psychiatric disorders and may or may not overlap between disorders [53]; and (iv) family members of patients with severe mental illness show similar brain deviations, albeit with smaller effect sizes [54]. Together with WP7, a multimodal dimension reduction approach to the study of psychiatric disorders will be applied to thousands of variables across different neuroimaging modalities, i.e., T1-weighted imaging, diffusion tensor imaging, and resting-state functional MRI. The predictive power of integrated multimodal components, in addition to ‘classical’ unimodal metrics (such as brain volume or functional connectivity strength), can be tested by *predicting* offspring's mental health problems later in life, taking measures of parental mental health into account, both in the general population and in families at high risk for mental illness. Moreover, multimodal components also aid biological interpretation across the various modalities. This will facilitate mechanistic understanding about which brain regions or structures are more likely to ‘work together’. In addition, we aim to, for the first time, examine the degree to which parental mental health problems during the offspring’s prenatal phase or during early childhood are associated with overlapping or distinct features of brain structure or function between ill parent, partner, and child. Recent research suggests that parent–child relationships influence children's brain development [55], while parental brain networks that are associated with bonding-related behaviour adapt when becoming a parent [56]. While suggestive, previous neuroimaging studies did not jointly examine the neural pathways of intergenerational transmission of mental health problems in parents and children. Strength of this trio modelling approach to the intergenerational transmission of brain developmental variations is that familial and genetic confounding can be controlled for.

Analyses will focus on brain metrics from childhood into young adulthood, linking parental mental illness to offspring mental health outcomes in the age range where symptoms may occur. Relevant predictors will then be tested for stability vs change across development using the available repeated MRI assessments in population cohorts and in the familial high-risk offspring cohorts (Table 2).

### WP6: Animal modelling to increase mechanistic understanding of risk transmission

In WP6, we aim to establish causal mechanisms of intergenerational transmission of risk by exploiting two different mouse models, thereby going beyond what is possible in humans. First, *environmentally induced* molecular and epigenetic changes in the germline will be investigated as possible route of transmission of disorder-like traits from mother to offspring. Inbred mouse models of risk for maladaptive behavioural responses and cognitive functions can determine to what extent molecular/epigenetic factors, maternal behaviours, and nurturing contribute to transmission of disorder traits to the offspring independently or synergistically. We will use a model of early postnatal life adversity known to cause severe behavioural and cognitive impairment in adulthood, and strategies to distinguish the effects of maternal behaviour from molecular/epigenetic factors in reproductive cells [57]. Quantitative and qualitative measures of maternal care during offspring postnatal development will be obtained from ‘mentally-ill’ females and molecular/epigenetic analyses will be conducted in oocytes and female reproductive tissue. To distinguish between prenatal and maternal factors after birth and assess the causal relationship between symptoms in mothers and symptoms in the offspring, embryo transfer will be conducted in a way to grow offspring from ‘mentally-ill’ females in a normal intrauterine environment and vice versa. Cross-fostering will also be utilised to assess the contribution of maternal behaviours. For translational perspectives, the effects of intervention by environmental enrichment will be examined to determine if disorder-like traits can be attenuated or corrected in mothers and if their transmission to the offspring can be prevented, as previously demonstrated for paternal trauma [58].

Second, we will dissect the behavioural and neuroanatomical signatures of genetic and maternal environment risk underlying the intergenerational transmission of psychosis-related behaviour using the 22q11 microdeletion (22q11.2DS) mouse model [59, 60]. Human adults with 22q11.2DS exhibit a range of behavioural, cognitive, and neuroanatomical alterations that put them at increased risk for psychosis. This mouse model will enable the distinction between *genetic transmission* and *genetic nurture* (see also Fig. 3). A 2 × 2 breeding design will be implemented with adult female and male mice carrying 22q11.2DS. The four resulting experimental groups are (i) wild type (WT) offspring raised by WT mothers (healthy control); (ii) WT offspring raised by 22q11.2DS mothers (maternal environmental risk, i.e., genetic nurture); (iii) 22q11.2DS offspring raised by WT mothers (genetic transmission); and (iv) 22q11.2DS offspring raised by 22q11.2DS mothers (genetic transmission and genetic nurture). This model allows investigation of quantitative and qualitative measures of genetic transmission and genetically driven maternal environmental risk by evaluating psychosis-related behaviour and cognition during offspring postnatal development. Again, cross-fostering will be utilised to assess causality.

Further integration of findings between the two mouse models will be implemented in parallel experiments to assess maternal behaviour during offspring postnatal development using maternal care measures.

### WP7: Multilevel, multimodal integration, causal pathway models, and normative risk prediction

WP7 wraps around the biological domain-specific WPs to integrate the different types of data and information to achieve two fundamental goals: (i) to facilitate interpretation in terms of potential mechanistic insights into intergenerational transmission of risk; and (ii) to improve prediction of mental health outcomes using all the information and individual variation, in ways that are transferable across cohorts and individuals. Recent advances in independent component analysis (ICA) have been specifically addressing the integration of high dimensional data: genomic ICA [61, 62] and linked ICA [63, 64]. *Genomic ICA* transforms genome-wide associations of thousands of (brain) traits into a smaller set of genomic components that essentially 'group' SNPs according to the similarity of their effects on different brain traits. Genomic ICA achieves efficient data reduction in a way that aids interpretation in terms of biological mechanisms that drive gene-brain associations [61]. The resultant components increase reproducibility of the GWAS signal [62], and can be applied to any new genotyped cohort or individual to calculate polygenic scores per component, creating a new set of variables of individual's loadings on each multivariate component (used in WP3). *Linked ICA* is applied for the meaningful data reduction and integration of multimodal voxel-wise neuroimaging data (used in WP5), but will in WP7 be extended to include a range of other biological, environmental, and cognitive-behavioural variables (i.e., ‘SuperBigFLICA’) for supervised data reduction [64]. Integrating association patterns across biological levels and modalities from human cohort data through genomic and linked ICA can reveal putative mechanisms: metrics and voxels loading highly on the same component are statistically dependent, and reflect joint biological processes [65].

**Fig. 4:**
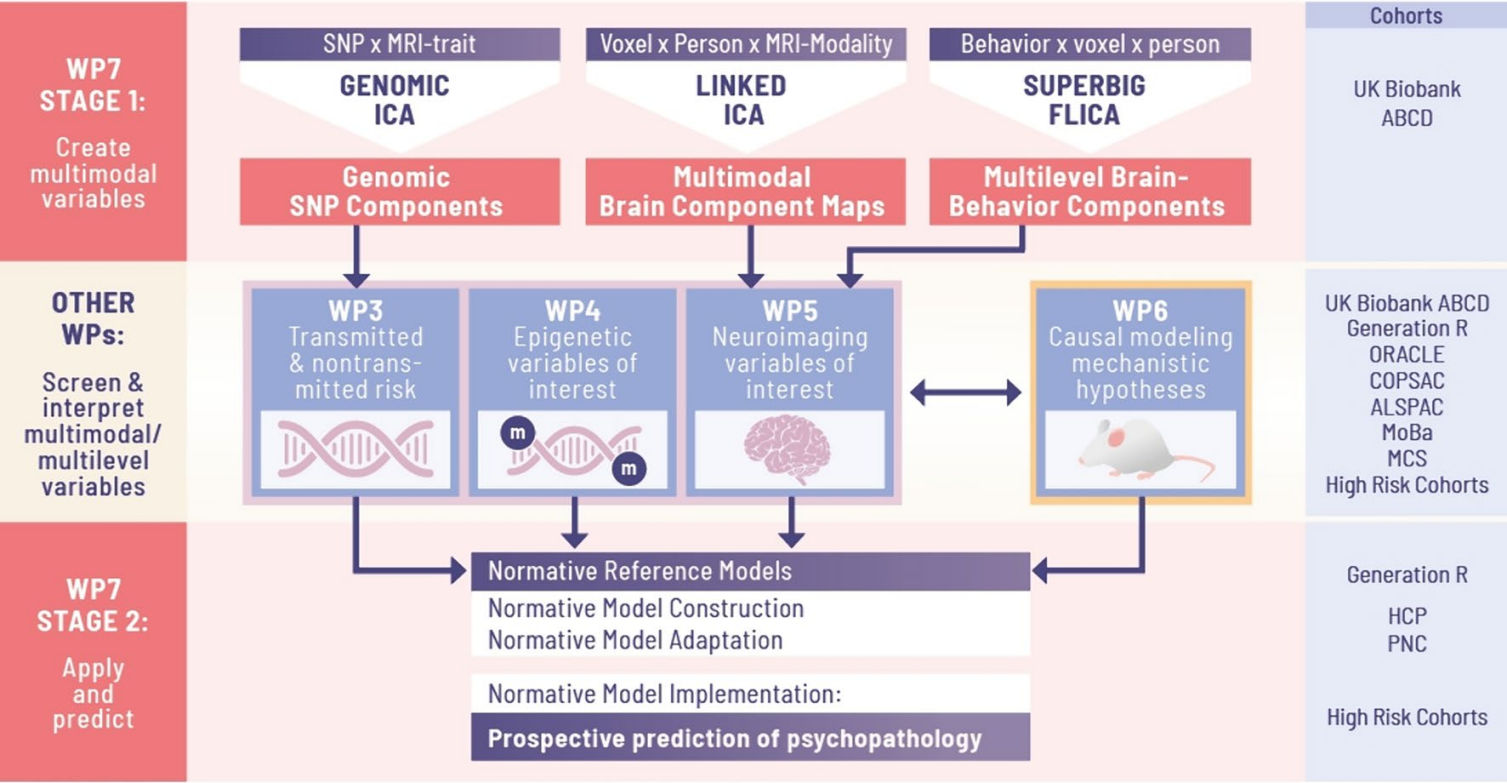
The work package 7 framework

In stage 1, WP7 will use a fully data-driven way to generate different types of multimodal components with putative predictive value from UK Biobank and ABCD data using both genetic and imaging data. These will be used as new, more interpretable, and more efficient machine-learning features in subsequent mechanistic as well as predictive analyses. To this end, these new feature sets will be passed on to WP3 (genetics) and WP5 (neuroimaging), to test their clinical relevance and potential predictive values in independent population and familial high-risk cohorts. In stage 2, clinically relevant markers from the specialised data domains (WPs3-5) and from the experimental testing of the hypothesised mechanisms in the animal (WP6) will be used to create, test, and generalise predictive models in and between the general population and offspring at high familial risk (Fig. 4); and to test putative mechanisms in dedicated causal pathway models. For example, the temporal ordering in the data, i.e., repeated assessments within individuals, allows for testing cross-lagged models that postulate pathways linking early exposures to later life health outcomes or examining how traits develop with increasing age within-person and assess time-dependent interrelationships. These analyses will integrate several biological levels (e.g., genetics, epigenetics, imaging, psychosocial) to build extended chains of risk transmission (mediation). Factors earlier identified as putative resilience factors will be entered as moderators, to test their risk-dampening effects.

Further, the results from WPs3-5 will be used by WP7 to develop normative prediction models where symptoms in offspring will be modelled as a function of predictive multimodal risk features along developmental trajectories and individual deviations from predicted risk. This allows for predicting the risk of symptoms in new (independent) individuals [66, 67]. The method builds age- and sex-specific ‘normative’ (i.e., average range reference) trajectories from relevant predictors, notably FAMILY's multilevel, multimodal, and genomic components from the ICAs. Given normative models, new out-of-sample individuals can then be characterised in terms of their deviation from those reference trajectories, while controlling for specific demographics. This may constitute either risk or resilience if symptoms are respectively higher or lower than predicted symptoms. Key advantages of FAMILY's novel normative modelling approach are that (i) the reference models can be flexibly adapted to the characteristics of different target populations by including different additional reference samples [67]; and (ii) it is sensitive to heterogeneity in different clinical populations because the deviation scores do not assume that all patients (or target group members) differ from the reference in a consistent way [68]. The deviation scores will be entered as predictors for prospective, future risk- and resilience estimation, in addition to psychological, clinical, and environmental factors. FAMILY will further develop the normative modelling approach towards more individualised realistic applications by implementing the family context.

### WP8: Ethical and social consequence of individualised risk prediction

Risk prediction can give rise to ethical and social issues, e.g. stigma, risk communication, and risk perception [69, 70]. Within a family context, additional issues arise, such as reproductive choices or the right of offspring not to know. Moreover, questions arise with respect to the ethical and professional duty of mental healthcare professionals to recognise and address risk and/or resilience indicators in families of their patients [71]. Although legal frameworks in different countries vary, ethical frameworks are likely to be consistent in requiring special safeguards for both offspring exposed to parental mental illness and parents suffering from mental illness. In WP8, we aim to systematically investigate how ethical and legal normative frameworks should be amended in the context of research and clinical settings. Specifically, cultural, age, and sex/gender aspects in relation to ethical and social consequences of risk prediction (e.g., stigma, shame, guilt) from neurobiological, psychological, environmental, and clinical information will be considered.

We will analyse ethical and normative issues in the context of intergenerational transmission of risk of mental illness and the use of prediction models. That is, we will reflect on how ‘structural discrimination’ exacerbates individual patients’ health problems [72]. Furthermore, empirical data will be gathered through qualitative (semi-structured interviews) and quantitative (survey) research methods, according to standards of practice for empirical bioethics research [73] across several European member states. These empirical data gathered will be integrated with ethical arguments to develop ethical guidelines for professionals to assist in the clinical use of prediction models, as well as empowerment of people with mental disorders and their families. Knowledge gained in WPs3-7 will be shared with WP8 and serve as input for the interviews and surveys.

### WP2: Data management and infrastructure for data sharing

Given the large number of data, WP2 is dedicated to guarantee data management in line with the Findable, Accessible, Interoperable and Reusable (FAIR)-principles and General Data Protection Regulation (GDPR)-compliant storage of and access to data sets. Standard Operating Procedures (SOPs) for data harmonisation (following the FAIR-principles), data-merging across, and data access among partner sites for all data types will be centrally developed within FAMILY. Harmonisation of datasets will leverage on existing efforts and plans already in place with large, EU-funded consortia using similar data types and structures (e.g. LifeCycles [74], Early Cause [75]) which jumpstarts the harmonisation process. Requests for data access will be supervised by a Data Access Committee based on the data access policy, which will be developed. The Data Access Committee will conduct scientific evaluation of proposals requesting data.

In FAMILY, all processing of data (when local regulations allow) will be implemented on a dedicated research infrastructure, the Digital Research Environment (DRE: https://www.andrea-cloud.eu/) following GDPR-standards. The DRE provides a secure, flexible, scalable cloud-based platform where researchers have access to and can work (together) with the data, methods, and models available in FAMILY. The DRE operates on the Microsoft Azure platform (which respects intellectual property rights), and the hardware is located within the EU. The architecture of the DRE allows researchers to use a solution within the boundaries of data management rules and regulations as will be put in place by WP2. To ensure the longevity of the FAMILY infrastructure, FAMILY will develop a long-term data (re)use model. Importantly, this strategy also allows for new groups to incorporate their data into the DRE, expanding the potential of this resource.

## Results

FAMILY's impact will be substantial in the areas of knowledge generation via open access scientific publications, code, and tool and method development (scientific impact), as well as in developing ethical guidelines and creating awareness on the impact of intergenerational transmission of risk of mental illness via non-specialised and national publications (societal and clinical impact). To maximise impact, FAMILY has devised dedicated strategies for disseminating and exploiting FAMILY results, e.g., through conferences, workshops, press releases, and trainings. These efforts will lead to dialogue and coordination between stakeholders (researchers, patients and their family members and mental healthcare professionals) and policymakers as well as integration across different settings (i.e., mental health services for children and adolescents and for adults). Specifically, by mapping social and ethical consequences of risk prediction (whether family-based) FAMILY will prepare the field for clinical implementation of risk prediction models. Additionally, FAMILY will implement communication strategies that aim to educate and empower citizens of all ages and throughout their life, by educating them on mental health problems, the latest scientific discoveries, and the role of intergenerational transmission to reduce stigma. Increased awareness and knowledge about transmission of risk from parent to offspring will support vulnerable families in taking an active role in the self-management of their own health. Moreover, insights and knowledge arising from FAMILY’s approach will provide relevant targets that allow future initiatives to design better strategies and personalised tools for preventing disorders and promoting health.

## Discussion

FAMILY’s key objectives are to improve causal understanding and gain prediction accuracy from the family context by the innovative combination of unique data collection, statistical modelling of genetically informed designs, causal inference, normative prediction, and animal modelling. FAMILY’s focus is on the mediating role of biological factors, such as epigenetic and brain markers, however, with our infrastructure, available data and methods, we can delineate genetic and non-genetic routes of transmissions and other mediators as well, such as behaviour, personality, life experiences, or social conditions. Furthermore, FAMILY will address key bioethical and social issues raised by the concept of intergenerational risk transmission and risk prediction. In the years to come, FAMILY will break new ground in understanding and predicting risk for and resilience against mental illness. Importantly, families will be considered as a source of information to fill critical knowledge gaps and allow the identification of the risk of transmission of mental illness from parents to offspring. FAMILY will further develop the normative modelling approach towards more individualised realistic applications by implementing the family context and providing answers to questions that are relevant to the individual, e.g.: What can I expect, given my family’s and my genetic background, and in the context of my family’s past and current circumstances? Which biological and environmental factors are most relevant for me, in terms of deciding on which risk-reduction and resilience-strengthening strategies will be most effective? In-depth causal analyses of how and when risk for mental illness occurs, will help identify early risk and resilience factors and predict who is likely to be diagnosed or develop symptoms of mental illness. Advanced insights can uncover new targets for the development of preventive strategies to break the intergenerational cycle of mental illness and to support strengths and resource building. An immediate benefit will be to open direct translational perspectives to mental healthcare professionals by contributing to new (family-based) risk prediction models for the early identification of adults and children at risk and by delivering ethical guidelines to guide its implementation. This will accelerate preventive and treatment intervention in vulnerable families and help target resilience strategies to prevent the transition from health to disorder despite high familial risk.

## Consent to participate

Free and voluntary informed consent to participate in the respective local study was initially obtained from all individual participants and/or their parents/legal guardians included in the cohorts that will be part of the FAMILY framework.

## Data Availability

Data from familial high risk cohorts will be available upon reasonable request, given the highly sensitive nature of our data (email: family@erasmusmc.nl).
